# Synthesis, crystal structure and thermal properties of di­aqua­bis­(4-cyano­pyridine)­dithiocyanato­nickel(II)

**DOI:** 10.1107/S2056989026003373

**Published:** 2026-04-10

**Authors:** Christian Näther

**Affiliations:** a Institut für Anorganische Chemie, Universität Kiel, Germany; University of Aberdeen, United Kingdom

**Keywords:** synthesis, crystal structure, nickel thio­cyanate, discrete aqua complex, 4-cyano­pyridine, hydrogen bonding, thermal properties

## Abstract

The reaction of Ni(NCS)_2_ and 4-cyano­pyridine (C_6_H_4_N_2_) in water leads to the formation of single crystals of the title compound, [Ni(NCS)_2_(C_6_H_4_N_2_)_2_(H_2_O)_2_]. The asymmetric unit consists of one Ni^II^ cation, which is located on a center of inversion, as well as one thio­cyanate anion, one 4-cyano­pyridine coligand and one water mol­ecule in general positions. The nickel cations therefore adopt *trans*-NiO_2_N_4_ octa­hedra, forming discrete complexes. These complexes are linked by O—H⋯S hydrogen bonds into layers that lie parallel to the *bc* plane. These layers are additionally linked by weak C—H⋯N inter­actions into a three-dimensional network. Powder X-ray diffraction (PXRD) indicates that an almost pure sample has been obtained. Upon heating, the title compound loses its water mol­ecules and the 4-cyano­pyridine ligands in separate steps.

## Abstract 2

In the crystal structure of the title compound, the nickel cations are octa­hedrally coordinated by two terminal N-bonding thio­cyanate anions, two 4-cyano­pyridine ligands and two water mol­ecules into discrete complexes that are linked by O—H⋯S hydrogen bonds into layers. These layers are further connected into a three-dimensional network by weak C—H⋯N inter­actions.

## 1. Chemical context

Investigations on the synthesis of new coordination compounds with specific magnetic properties is still an important field in inorganic chemistry (Ferrando-Soria *et al.*, 2017 ▸). In this context, compounds in which paramagnetic metal cations are linked by small sized anionic ligands are of special importance (Yue & Gao, 2019 ▸). Numerous examples of such compounds are reported in the literature and our own work has focused on the synthesis of transition-metal thio­cyanate compounds in which the cations are linked by μ-1,3-bridging anionic ligands (Wöhlert *et al.*, 2013 ▸; Werner *et al.*, 2014 ▸; Neumann *et al.*, 2018 ▸). Unfortunately, with less chalcophilic metal cations, the synthesis in solution mostly leads to compounds in which the thio­cyanate anions are only terminally coordinated to the N atom.

Therefore, many years ago we developed a new route, which is based on thermal ligand removal from simple precursor complexes (Näther & Greve, 2003 ▸). We also have found that the corresponding coordination polymers with seleno­cyanate can be prepared by this route (Wriedt & Näther, 2010 ▸; Wöhlert *et al.*, 2012 ▸). This route is therefore one more alternative to the preparation of new coordination compounds by typical solid-state methods such as mol­ecular milling (Braga *et al.*, 2005 ▸, 2006 ▸, James *et al.*, 2012 ▸; Do & Friščić, 2017 ▸; Stolar *et al.*, 2017 ▸) or reactions in melts (Müller-Buschbaum, 2005 ▸; Höller & Müller-Buschbaum, 2008 ▸; Zurawski *et al.*, 2012 ▸).

In view of magnetic properties, compounds based on Co(NCS)_2_ and Ni(NCS)_2_ are of special inter­est, because the former can show one-dimensional ferromagnetic ordering if they exhibit chain structures (Mautner *et al.*, 2018 ▸; Rams *et al.*, 2020 ▸), whereas the latter show three-dimensional ferromagnetic ordering if they crystallize as layered structures (Suckert *et al.*, 2016 ▸). In the course of this project, we were particularly inter­ested in pyridine derivatives as ligands and within our systematic investigations we became inter­ested in Ni(NCS)_2_ compounds with 4-cyano­pyridine (C_6_H_4_N_2_) as coligand.

With this ligand, only one Ni compound is reported: this is Ni(NCS)_2_(C_6_H_4_N_2_)_4_, which consists of discrete complexes in which the nickel cations are coordinated by two terminal N-bonded thio­cyanate anions and four 4-cyano­pyridine ligands, which coordinate through the pyridine N atom to the metal center (CSD refcode UBUBOL; Clegg & Harrington, 2016 ▸). In the course of our investigations, we obtained crystals of a further crystalline phase, Ni(NCS)_2_(C_6_H_4_N_2_)_2_(H_2_O)_2_ (**I**) that was identified by single crystal X-ray diffraction.
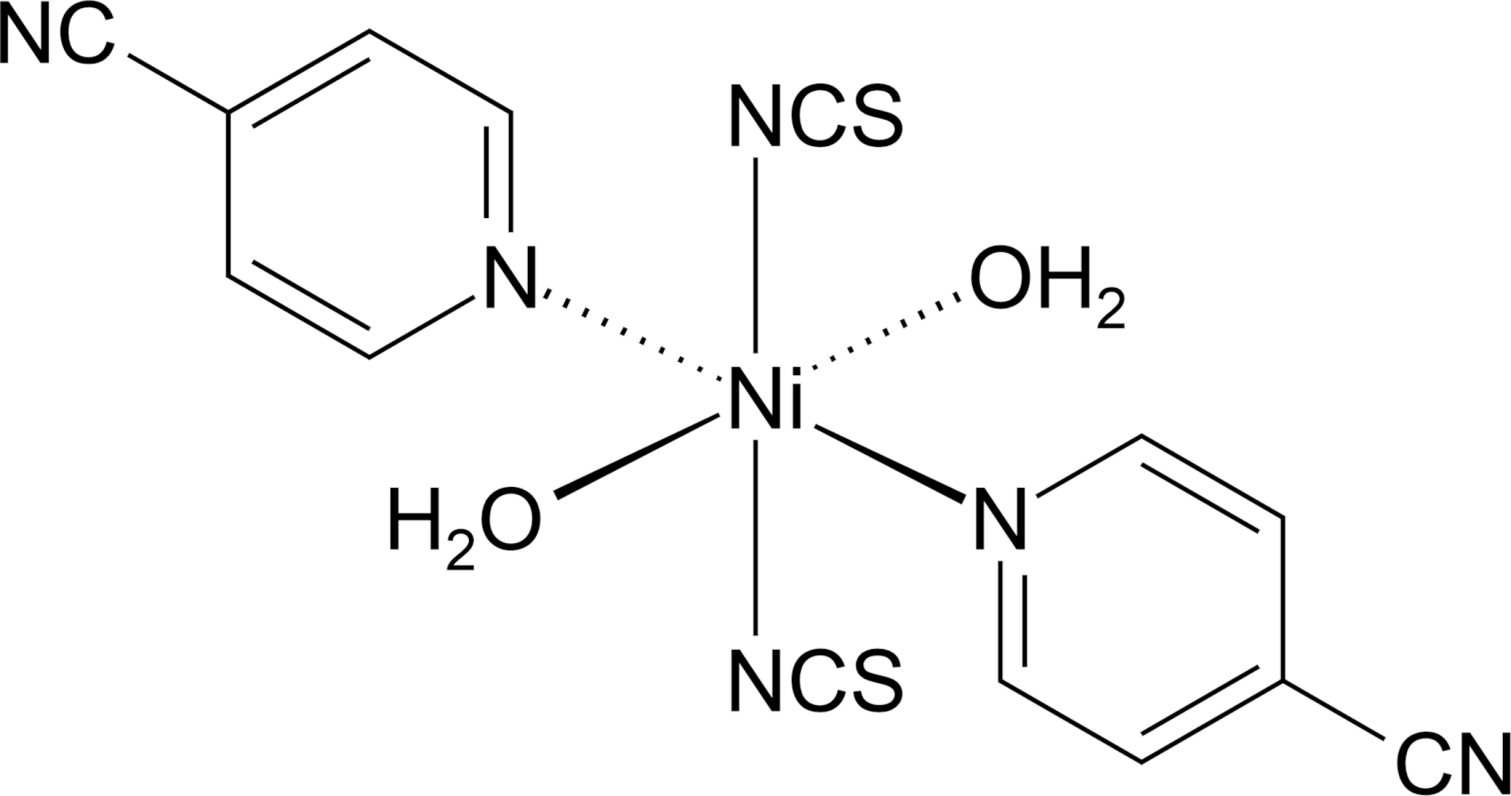


## 2. Structural commentary

**Figure 1:**
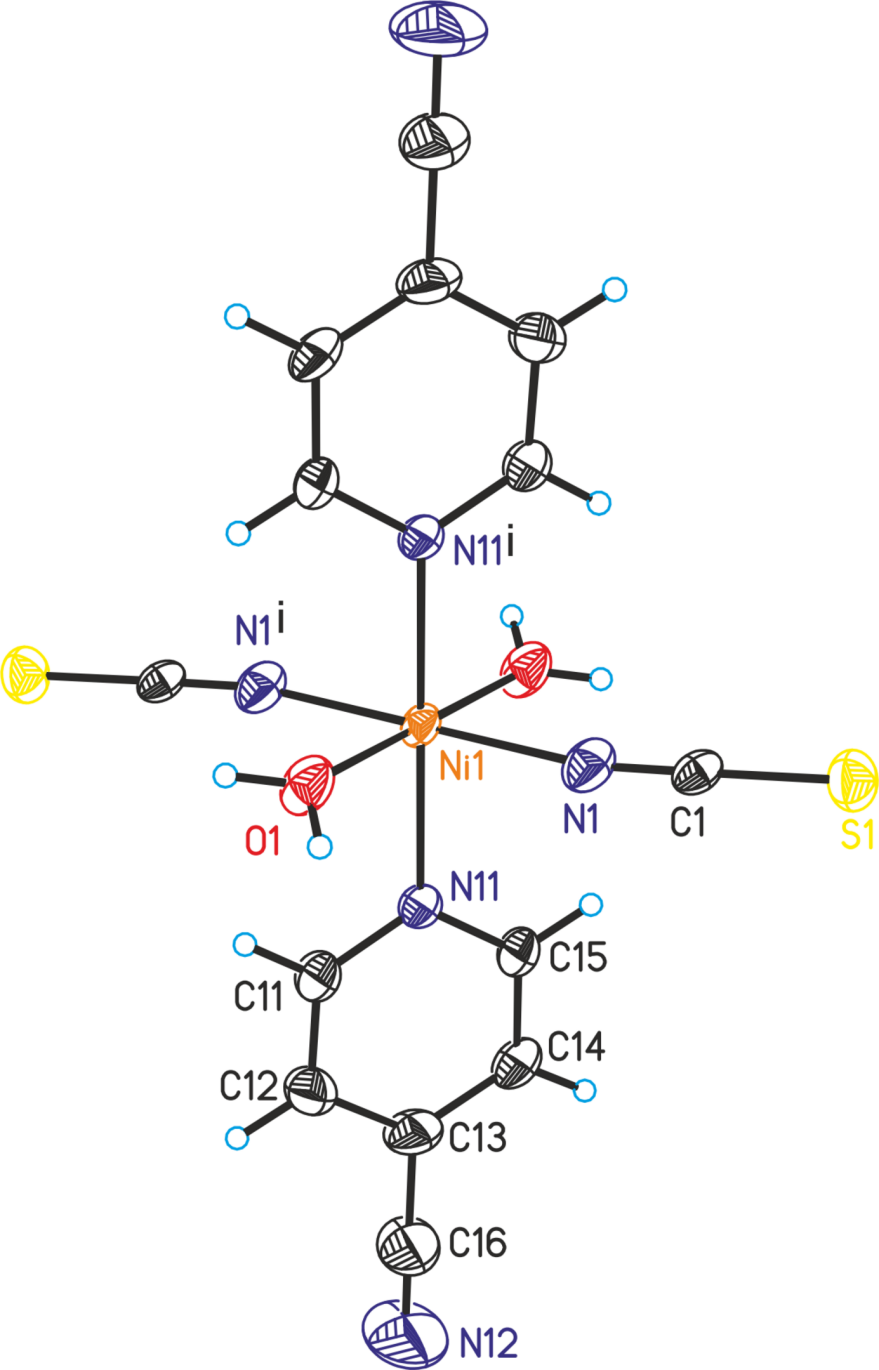
The mol­ecular structure of (**I**) with labeling and displacement ellipsoids drawn at the 50% probability level. Symmetry code: (i) −*x* + 1, −*y* + 1, −*z* + 1.

The asymmetric unit of (**I**) is built up of one crystallographically independent nickel(II) cation that is situated on a center of inversion and one thio­cyanate anion, one 4-cyano­pyridine coligand and one water mol­ecule, with all atoms located in general positions. The nickel cations are sixfold coordinated by two terminally N-bonding thio­cyanate anions, two 4-cyano­pyridine coligands and two water mol­ecules into discrete complexes (Fig. 1 ▸). The 4-cyano­pyridine coligand coordinates through its pyridine N atom to the metal center. The bond angles deviate slightly from the ideal values, which demonstrate that the *trans*-NiO_2_N_4_ octa­hedra are slightly distorted (Table 1 ▸).

Finally, it may be mentioned that the title compound is isotypic to one of the two modifications of Mn(NCS)_2_(C_6_H_4_N_2_)_2_(H_2_O)_2_ already reported in the literature (OJEFAN; Wellm *et al.*, 2020 ▸).

## 3. Supra­molecular features

**Figure 2:**
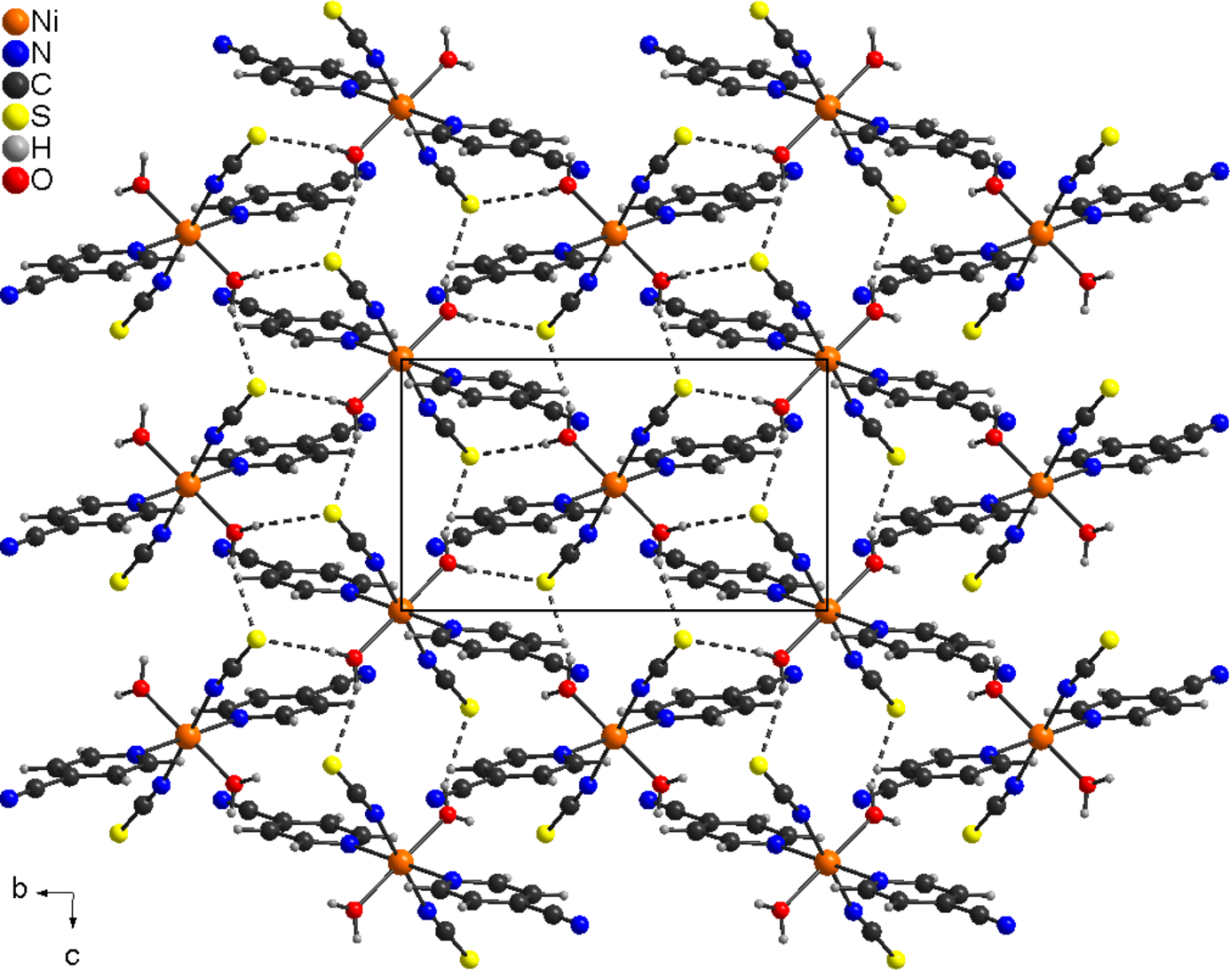
Crystal structure of (**I**) in a view along the crystallographic *a*-axis direction. The O—H⋯S hydrogen bonds are shown as dashed lines.

In the extended structure of (**I**), the complexes are linked by O—H⋯S hydrogen bonds between the water H atoms and the thio­cyanate S atom into corrugated layers that are arranged parallel to the *bc* plane (Fig. 2 ▸). Within these layers, each S atom acts as acceptor for two hydrogen bonds from two neighboring complexes, and the H atoms of each water mol­ecule act as donors in O—H⋯S hydrogen bonds to two complexes. Therefore, each complex is involved in eight hydrogen bonds to six adjacent complexes (Fig. 2 ▸). The H⋯S distances are relatively short and the O—H⋯S angles close to linear, indicating that these are relatively strong inter­actions (Table 2 ▸).

**Figure 3:**
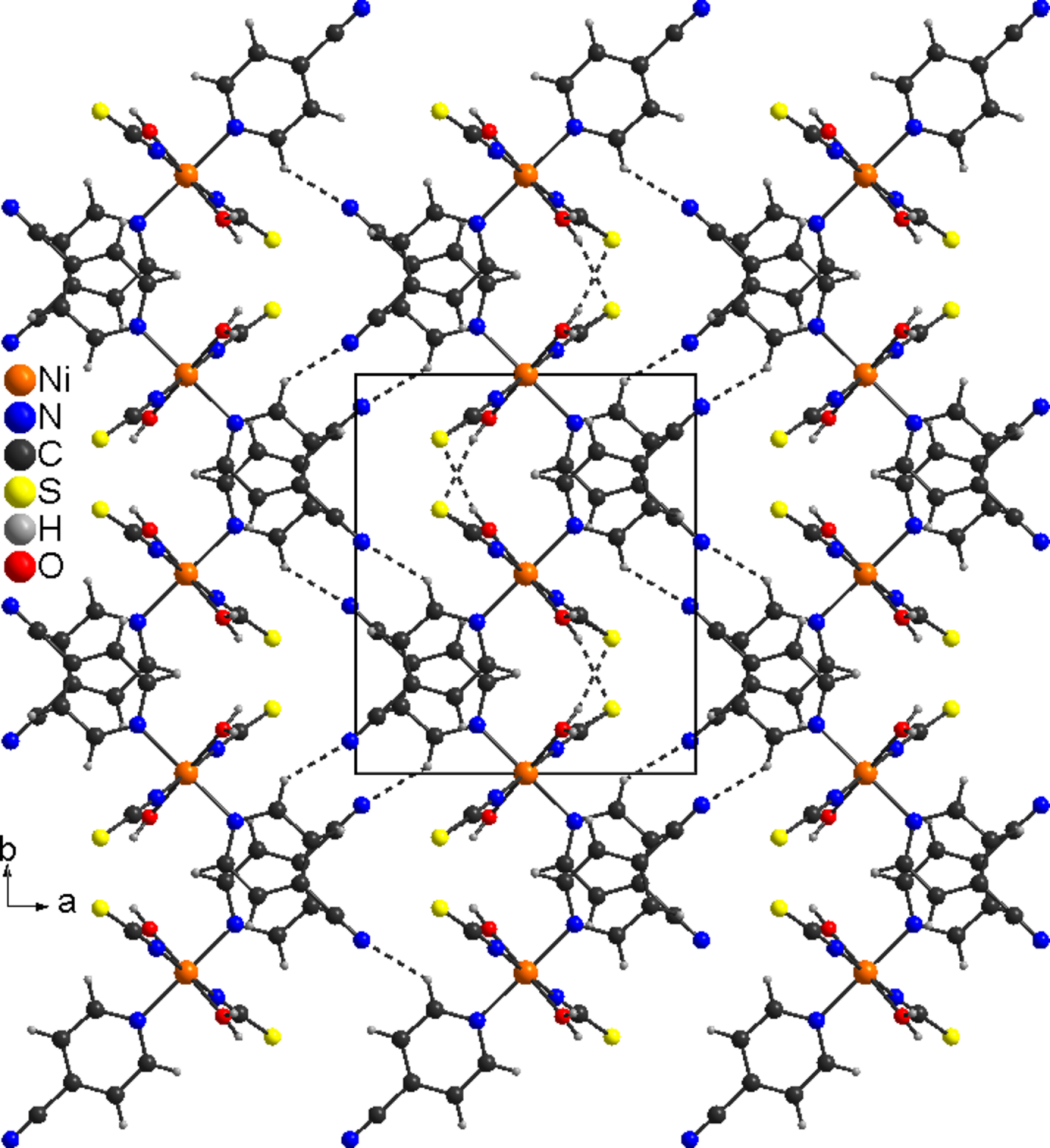
Crystal structure of (**I**) in a view along the crystallographic *c*-axis direction. Inter­molecular O—H⋯S and C—H⋯N hydrogen bonding is shown as dashed lines.

The layers are additionally linked by pairs of centrosymmetric C—H⋯N inter­actions between the pyridine H atom and the N atom of the cyano group into a three-dimensional network (Fig. 3 ▸). The C—H⋯N angle is far from linear, showing that this is only a weak inter­action (Table 2 ▸).

## 4. Additional characterization

**Figure 4:**
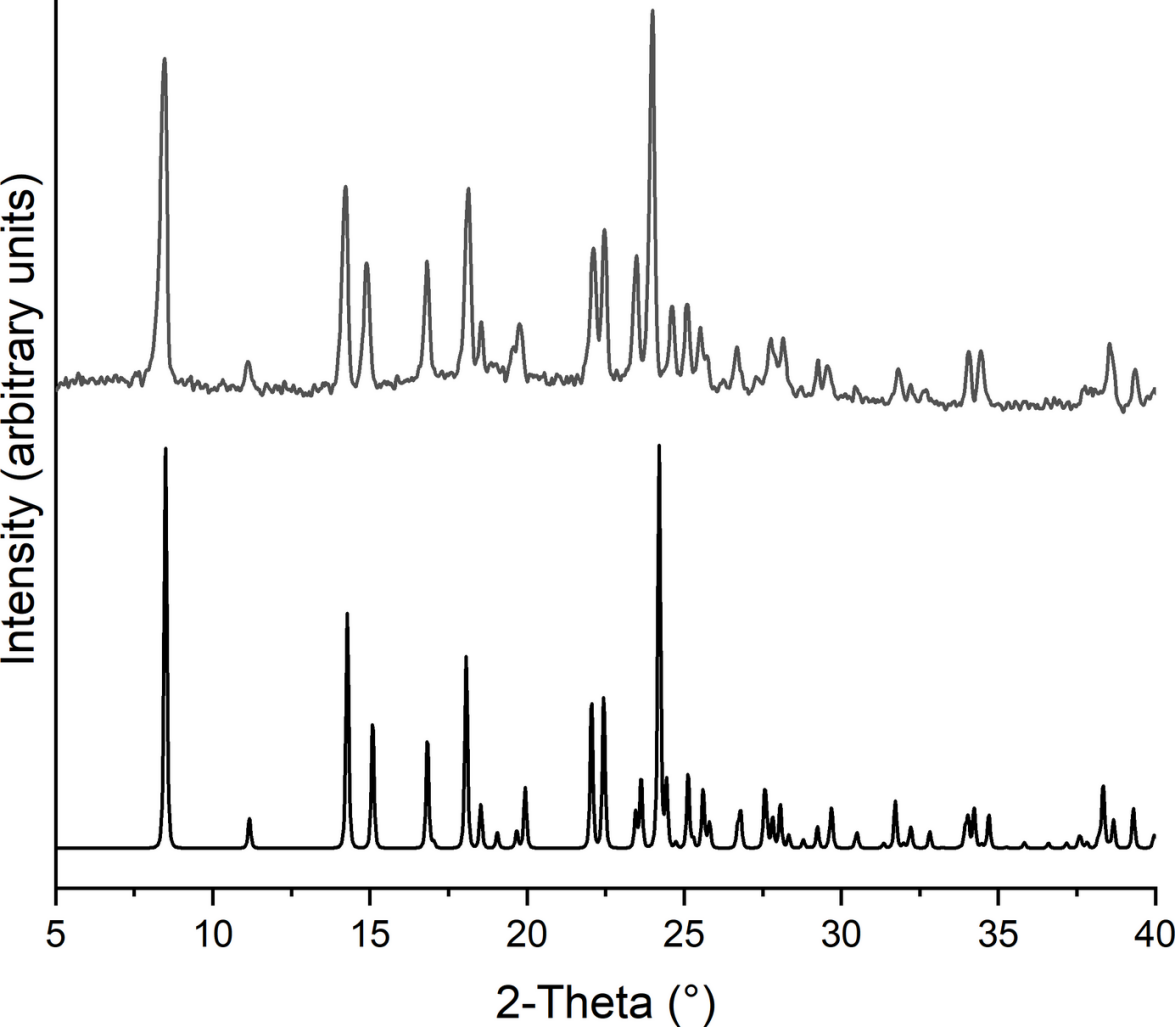
Experimental (top) and calculated (bottom) PXRD patterns for (**I**). Some of the reflections in the calculated pattern are slightly shifted to higher Bragg angles, which originate from the fact that the structure analysis was performed at lower temperatures.

The purity of (**I**) was investigated by X-ray powder diffraction. Comparison of the experimental powder pattern with that calculated for the title compound proves that an almost pure crystalline phase has been obtained (Fig. 4 ▸). There is a very small additional peak to the left of the most intense reflection, which indicates the presence of a very small amount of a further crystalline phase. The experimental pattern is rather noisy, which can be traced back to a low crystallinity, presumably because of grinding.

**Figure 5:**
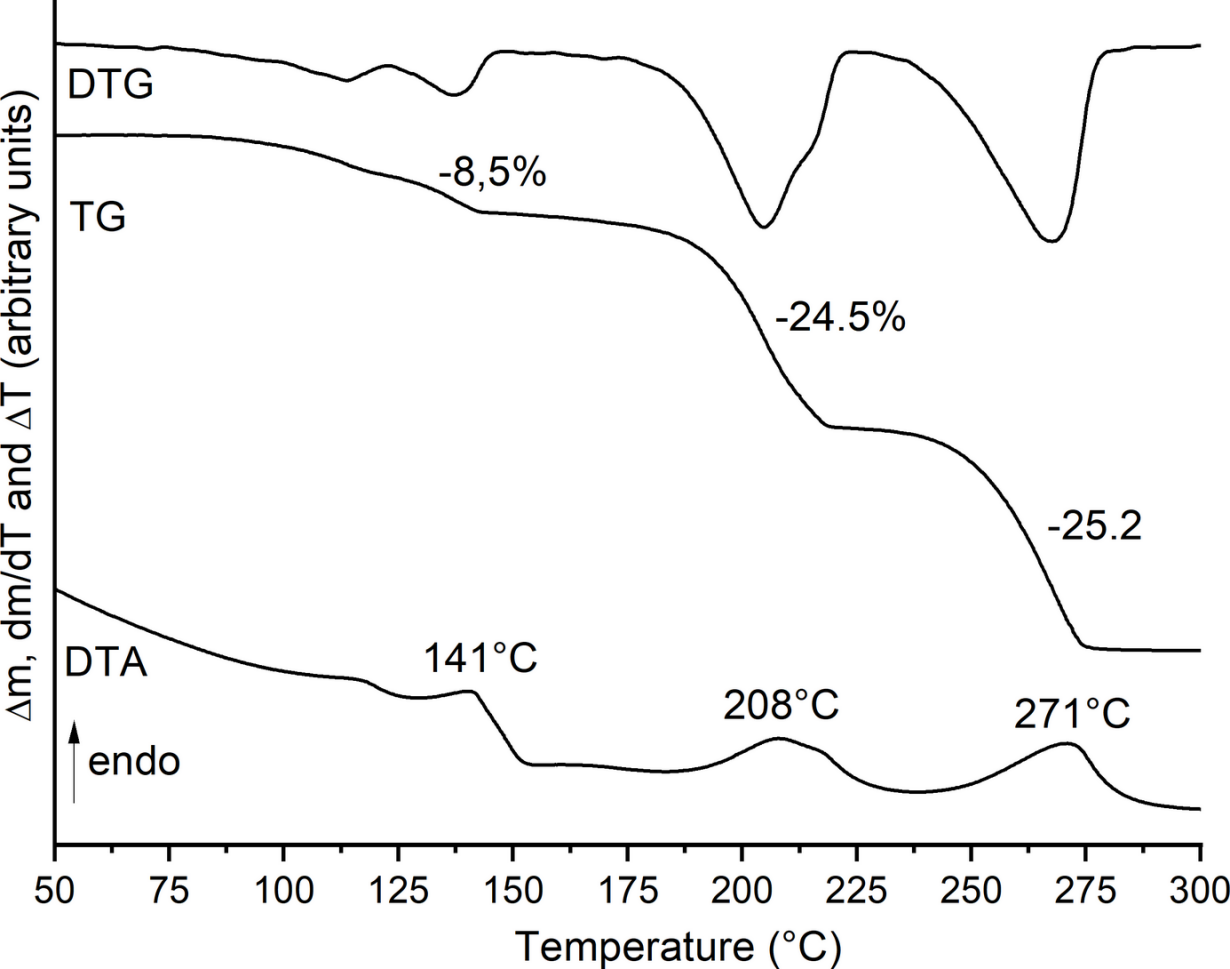
DTG, TG and DTA curves for (**I**). The percentage mass loss is given and the peak temperature is in °C.

The thermal properties of (**I**) were investigated using differential thermoanalysis coupled to thermogravimetry (DTA-TG). Upon heating, at least three mass losses are observed in the TG curve that are accompanied with endothermic events in the DTA curve (Fig. 5 ▸). The experimental mass loss in the first step of 8.5% is in good agreement with that calculated for the removal of the two water mol­ecules of 8.1%. The DTG curve indicates that the first mass loss can be divided into two different steps, which is a hint that the two water mol­ecules are removed in two different steps. The experimental mass losses in the second and third TG step are in good agreement with those calculated for one 4-cyano­pyridine ligand in each step (Δ*m*_calc._ = 24.8%). This indicates that in the first TG step a compound with the composition Ni(NCS)_2_(C_6_H_4_N_2_)_2_ is formed, which decomposes into Ni(NCS)_2_(C_6_H_4_N_2_) upon further heating, before the final product of Ni(NCS)_2_ is formed.

## 5. Synthesis and characterization

Ni(NCS)_2_ was prepared from the reaction of equimolar amounts of NiSO_4_·6H_2_O with Ba(NCS)_2_·3H2O in water. The white residue of BaSO_4_ was filtered off and the filtrate was concentrated until complete dryness. The purity was checked by X-ray powder diffraction (XRPD). Barium thio­cyanate trihydrate was purchased from Alfa Aesar and 4-cyano­pyridine as well as nickel sulfate hexa­hydrate from Sigma-Aldrich.

1.00 mmol (174.9 mg) of Ni(NCS)_2_ and 2.00 mmol (208.2 mg) of 4-cyano­pyridine were reacted in 3 ml of water at room temperature. Within 3 d, crystals of (**I**) in the form of light-blue blocks suitable for crystal structure analysis were obtained.

Powder X-ray diffraction measurements were performed using a Stoe STADI P transmission powder diffractometer with Cu *K*α_1_ radiation (λ = 1.540598 Å), a Johann-type Ge(111) monochromator and a MYTHEN 1K detector from Dectris.

Thermogravimetry and differential thermoanalysis (TG–DTA) measurements were performed in a dynamic nitro­gen atmosphere in Al_2_O_3_ crucibles with a heating rate of 4°C min^−1^ using a STA-PT 1000 thermobalance from Linseis. The TG–DTA instrument was calibrated using standard reference materials.

## 6. Database survey

Some more compounds with 4-cyano­pyridine and transition-metal cations were found in a search of the CSD (version 5.43, last update January 2026; Groom *et al.*, 2016 ▸) using CONQUEST (Bruno *et al.*, 2002 ▸). These include Ni(NCS)_2_(C_6_H_4_N_2_)_4_ already mentioned in the *Chemical context* section (CSD refcode UBUBOL; Clegg & Harrington, 2016 ▸).

Several compounds are reported with Mn(NCS)_2_, including Mn(NCS)_2_(C_6_H_4_N_2_)_4_, that also consists of discrete complexes, but which are not isotypic to Ni(NCS)_2_(C_6_H_4_N_2_)_4_ (OJEDOZ; Wellm *et al.*, 2020 ▸). Discrete complexes with an octa­hedral coordination are also found in one of the two modifications of Mn(NCS)_2_(C_6_H_4_N_2_)_2_(H_2_O)_2_ that is isotypic to the title compound (OJEFAN and OJEFAN01; Wellm *et al.*, 2020 ▸). Two further aqua complexes with additional 4-cyano­pyridine as solvate ligand are also known (OJEFER and OJEFUH; Wellm *et al.*, 2020 ▸). Two Mn(NCS)_2_ compounds with bridging thio­cyanate anions are also reported. These include Mn(NCS)_2_(C_6_H_4_N_2_)_2_, in which the manganese cations are octa­hedrally coordinated by two N- and two S-bonding thio­cyanate anions and two 4-cyano­pyridine ligands and are linked by pairs of anionic ligands into chains (OJEFIV; Wellm *et al.*, 2020 ▸) and Mn(NCS)_2_(C_6_H_4_N_2_), in which two Mn^II^ cations are linked by pairs of thio­cyanate anions into dinuclear units that are further connected by single μ-1,3-bridging anionic ligands into layers that condense into a three-dimensional network *via* the bridging 4-cyano­pyridine ligands (OJEDUF and OJEDUF01; Wellm *et al.*, 2020 ▸).

With Cu(NCS)_2_, one compound with the composition Cu(NCS)_2_(C_6_H_4_N_2_)_2_ is also found, in which the 4-cyano­pyridine coligand is only monocoordinated *via* the pyridine N atom and in which the copper cations are linked by pairs of thio­cyanate anions into chains (ABOVOF; Handy *et al.*, 2017 ▸). A further solvato complex with the composition Fe(NCS)_2_(C_6_H_4_N_2_)_2_(H_2_O)_2_·2(C_6_H_4_N_2_) is also known (Jochim *et al.*, 2017 ▸).

Finally, two compounds with Cd(NCS)_2_ are listed in the CSD, *viz*. Cd(NCS)_2_(C_6_H_4_N_2_)_2_ (WUCLUB; Chen *et al.*, 2002 ▸), which shows the same structure as Mn(NCS)_2_(C_6_H_4_N_2_)_2_, and Cu(NCS)_2_(C_6_H_4_N_2_)_2_ (WUCMAI; Chen *et al.*, 2002 ▸), in which the 4-cyano­pyridine ligand acts as a bridging ligand.

## 7. Refinement

Crystal data, data collection and structure refinement details are summarized in Table 3 ▸. The C-bound H atoms were positioned with idealized geometry and were refined with *U*_iso_(H) = 1.2*U*_eq_(C) using a riding model. The water H atoms were located in a difference map, their bond lengths were set to standard values and finally they were refined with *U*_iso_(H) = 1.5*U*_eq_(O) using a riding model. The crystal chosen for data collection was found to contain a small amount of at least a second domain, but it was not possible to index them separately to perform a twin refinement.

## Supplementary Material

Crystal structure: contains datablock(s) I. DOI: 10.1107/S2056989026003373/hb8207sup1.cif

Structure factors: contains datablock(s) I. DOI: 10.1107/S2056989026003373/hb8207Isup2.hkl

CCDC reference: 2542643

Additional supporting information:  crystallographic information; 3D view; checkCIF report

## Figures and Tables

**Table 1:** Selected geometric parameters (Å, °)

Ni1—N1	2.035 (3)	Ni1—N11	2.108 (3)
Ni1—O1	2.084 (2)		
			
N1—Ni1—O1^i^	87.62 (12)	N1—Ni1—N11	89.67 (13)
N1—Ni1—O1	92.38 (12)	O1—Ni1—N11	91.15 (11)
N1^i^—Ni1—N11	90.32 (13)	C1—N1—Ni1	166.4 (3)

Symmetry code: (i) 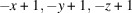.

**Table 2:** Hydrogen-bond geometry (Å, °)

*D*—H⋯*A*	*D*—H	H⋯*A*	*D*⋯*A*	*D*—H⋯*A*
O1—H1*O*1⋯S1^ii^	0.84	2.48	3.307 (3)	167
O1—H2*O*1⋯S1^iii^	0.84	2.44	3.225 (3)	156
C11—H11⋯N12^iv^	0.95	2.44	3.129 (6)	129

Symmetry codes: (ii) 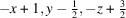; (iii) 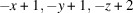; (iv) 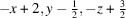.

**Table 3:** Experimental details

Crystal data	
Chemical formula	[Ni(NCS)_2_(C_6_H_4_N_2_)_2_(H_2_O)_2_]
*M*_r_	419.13
Crystal system, space group	Monoclinic, *P*2_1_/*c*
Temperature (K)	200
*a*, *b*, *c* (Å)	10.6984 (11), 12.2068 (10), 7.3974 (7)
β (°)	103.500 (12)
*V* (Å^3^)	939.36 (16)
*Z*	2
Radiation type	Mo *K*α
μ (mm^−1^)	1.27
Crystal size (mm)	0.13 × 0.09 × 0.06

Data collection	
Diffractometer	Stoe IPDS1
Absorption correction	Numerical (*X-SHAPE* and *X-RED32*; Stoe, 2008 ▸)
*T*_min_, *T*_max_	0.844, 0.899
No. of measured, independent and observed [*I* > 2σ(*I*)] reflections	5054, 1830, 1464
*R*_int_	0.074
(sin θ/λ)_max_ (Å^−1^)	0.617

Refinement	
*R*[*F*^2^ > 2σ(*F*^2^)], *wR*(*F*^2^), *S*	0.055, 0.150, 1.02
No. of reflections	1830
No. of parameters	115
H-atom treatment	H-atom parameters constrained
Δρ_max_, Δρ_min_ (e Å^−3^)	1.24, −1.09

Computer programs: *X-AREA* (Stoe, 2008 ▸), *SHELXT* (Sheldrick, 2015*b* ▸), *SHELXL* (Sheldrick, 2015*a* ▸), *DIAMOND* (Brandenburg, 1999 ▸), *XP* in *SHELXTL-PC* (Sheldrick, 2008 ▸) and *publCIF* (Westrip, 2010 ▸).

## supplementary crystallographic information

### Diaquabis(pyridine-4-carbonitrile)dithiocyanatonickel(II) Crystal data

**Table:**

[Ni(NCS)_2_(C_6_H_4_N_2_)_2_(H_2_O)_2_]	*F*(000) = 428
*M**_r_* = 419.13	*D*_x_ = 1.482 Mg m^−^^3^
Monoclinic, *P*2_1_/*c*	Mo *K*α radiation, λ = 0.71073 Å
*a* = 10.6984 (11) Å	Cell parameters from 6692 reflections
*b* = 12.2068 (10) Å	θ = 4.6–26.9°
*c* = 7.3974 (7) Å	µ = 1.27 mm^−^^1^
β = 103.500 (12)°	*T* = 200 K
*V* = 939.36 (16) Å^3^	Block, light blue
*Z* = 2	0.13 × 0.09 × 0.06 mm

### Diaquabis(pyridine-4-carbonitrile)dithiocyanatonickel(II) Data collection

**Table:**

Stoe IPDS-1 diffractometer	1464 reflections with *I* > 2σ(*I*)
Phi scans	*R*_int_ = 0.074
Absorption correction: numerical (X-Shape and X-Red32; Stoe, 2008)	θ_max_ = 26.0°, θ_min_ = 2.6°
*T*_min_ = 0.844, *T*_max_ = 0.899	*h* = −13→12
5054 measured reflections	*k* = −13→15
1830 independent reflections	*l* = −9→9

### Diaquabis(pyridine-4-carbonitrile)dithiocyanatonickel(II) Refinement

**Table:**

Refinement on *F*^2^	Primary atom site location: dual
Least-squares matrix: full	Hydrogen site location: mixed
*R*[*F*^2^ > 2σ(*F*^2^)] = 0.055	H-atom parameters constrained
*wR*(*F*^2^) = 0.150	*w* = 1/[σ^2^(*F*_o_^2^) + (0.1103*P*)^2^] where *P* = (*F*_o_^2^ + 2*F*_c_^2^)/3
*S* = 1.02	(Δ/σ)_max_ < 0.001
1830 reflections	Δρ_max_ = 1.24 e Å^−^^3^
115 parameters	Δρ_min_ = −1.09 e Å^−^^3^
0 restraints	

### Diaquabis(pyridine-4-carbonitrile)dithiocyanatonickel(II) Special details

**Table:**

Geometry. All esds (except the esd in the dihedral angle between two l.s. planes) are estimated using the full covariance matrix. The cell esds are taken into account individually in the estimation of esds in distances, angles and torsion angles; correlations between esds in cell parameters are only used when they are defined by crystal symmetry. An approximate (isotropic) treatment of cell esds is used for estimating esds involving l.s. planes.

### Diaquabis(pyridine-4-carbonitrile)dithiocyanatonickel(II) Fractional atomic coordinates and isotropic or equivalent isotropic displacement parameters (Å^2^)

**Table:**

	*x*	*y*	*z*	*U*_iso_*/*U*_eq_	
Ni1	0.500000	0.500000	0.500000	0.0212 (2)	
N1	0.4116 (3)	0.5615 (3)	0.6935 (4)	0.0322 (7)	
C1	0.3433 (3)	0.6039 (3)	0.7731 (4)	0.0228 (7)	
S1	0.24664 (9)	0.66118 (9)	0.88587 (14)	0.0356 (3)	
N11	0.6437 (3)	0.6209 (2)	0.5724 (4)	0.0259 (7)	
C11	0.7680 (3)	0.5930 (3)	0.6064 (6)	0.0324 (8)	
H11	0.788824	0.517784	0.598705	0.039*	
C12	0.8678 (4)	0.6673 (3)	0.6520 (6)	0.0384 (10)	
H12	0.954934	0.644175	0.676692	0.046*	
C13	0.8361 (4)	0.7766 (3)	0.6604 (6)	0.0398 (10)	
C14	0.7090 (4)	0.8075 (3)	0.6257 (6)	0.0375 (9)	
H14	0.686068	0.882410	0.630713	0.045*	
C15	0.6155 (4)	0.7275 (3)	0.5833 (6)	0.0304 (8)	
H15	0.527780	0.748690	0.561004	0.036*	
C16	0.9377 (5)	0.8572 (4)	0.7069 (9)	0.0598 (15)	
N12	1.0171 (5)	0.9193 (5)	0.7477 (11)	0.099 (2)	
O1	0.6085 (3)	0.3904 (2)	0.6893 (4)	0.0352 (7)	
H1O1	0.654313	0.339361	0.663891	0.053*	
H2O1	0.639203	0.397151	0.804131	0.053*	

### Diaquabis(pyridine-4-carbonitrile)dithiocyanatonickel(II) Atomic displacement parameters (Å^2^)

**Table:**

	*U*^11^	*U*^22^	*U*^33^	*U*^12^	*U*^13^	*U*^23^
Ni1	0.0251 (4)	0.0130 (3)	0.0255 (4)	0.0010 (2)	0.0063 (2)	−0.0014 (2)
N1	0.0400 (17)	0.0227 (16)	0.0361 (17)	−0.0037 (13)	0.0131 (14)	−0.0042 (13)
C1	0.0307 (17)	0.0184 (16)	0.0207 (16)	−0.0058 (13)	0.0085 (14)	−0.0014 (13)
S1	0.0338 (5)	0.0416 (6)	0.0342 (5)	0.0045 (4)	0.0135 (4)	−0.0029 (4)
N11	0.0255 (14)	0.0164 (14)	0.0342 (16)	0.0005 (11)	0.0036 (12)	−0.0023 (11)
C11	0.0315 (19)	0.0180 (17)	0.046 (2)	0.0023 (14)	0.0060 (16)	−0.0020 (16)
C12	0.0284 (18)	0.029 (2)	0.053 (2)	−0.0004 (16)	0.0007 (17)	−0.0036 (18)
C13	0.038 (2)	0.026 (2)	0.052 (2)	−0.0102 (16)	0.0027 (18)	−0.0071 (18)
C14	0.043 (2)	0.0168 (17)	0.051 (2)	0.0001 (16)	0.0069 (18)	−0.0087 (16)
C15	0.0303 (17)	0.0194 (17)	0.041 (2)	0.0030 (14)	0.0078 (15)	−0.0047 (15)
C16	0.044 (3)	0.033 (2)	0.098 (4)	−0.007 (2)	0.007 (3)	−0.012 (3)
N12	0.057 (3)	0.052 (3)	0.176 (7)	−0.028 (2)	0.003 (3)	−0.021 (4)
O1	0.0470 (16)	0.0237 (13)	0.0311 (14)	0.0093 (12)	0.0017 (12)	−0.0001 (11)

### Diaquabis(pyridine-4-carbonitrile)dithiocyanatonickel(II) Geometric parameters (Å, º)

**Table:**

Ni1—N1^i^	2.035 (3)	C11—H11	0.9500
Ni1—N1	2.035 (3)	C12—C13	1.382 (6)
Ni1—O1^i^	2.084 (2)	C12—H12	0.9500
Ni1—O1	2.084 (2)	C13—C14	1.376 (6)
Ni1—N11^i^	2.108 (3)	C13—C16	1.446 (6)
Ni1—N11	2.108 (3)	C14—C15	1.381 (5)
N1—C1	1.161 (5)	C14—H14	0.9500
C1—S1	1.630 (4)	C15—H15	0.9500
N11—C11	1.339 (5)	C16—N12	1.127 (6)
N11—C15	1.342 (5)	O1—H1O1	0.8400
C11—C12	1.381 (6)	O1—H2O1	0.8400
			
N1^i^—Ni1—N1	180.0	N11—C11—C12	123.8 (4)
N1^i^—Ni1—O1^i^	92.38 (12)	N11—C11—H11	118.1
N1—Ni1—O1^i^	87.62 (12)	C12—C11—H11	118.1
N1^i^—Ni1—O1	87.62 (12)	C11—C12—C13	117.5 (4)
N1—Ni1—O1	92.38 (12)	C11—C12—H12	121.3
O1^i^—Ni1—O1	180.0	C13—C12—H12	121.3
N1^i^—Ni1—N11^i^	89.68 (13)	C14—C13—C12	119.9 (4)
N1—Ni1—N11^i^	90.33 (13)	C14—C13—C16	120.9 (4)
O1^i^—Ni1—N11^i^	91.15 (11)	C12—C13—C16	119.2 (4)
O1—Ni1—N11^i^	88.85 (11)	C13—C14—C15	118.7 (4)
N1^i^—Ni1—N11	90.32 (13)	C13—C14—H14	120.6
N1—Ni1—N11	89.67 (13)	C15—C14—H14	120.6
O1^i^—Ni1—N11	88.85 (11)	N11—C15—C14	122.6 (3)
O1—Ni1—N11	91.15 (11)	N11—C15—H15	118.7
N11^i^—Ni1—N11	180.00 (11)	C14—C15—H15	118.7
C1—N1—Ni1	166.4 (3)	N12—C16—C13	178.2 (8)
N1—C1—S1	178.9 (3)	Ni1—O1—H1O1	126.0
C11—N11—C15	117.5 (3)	Ni1—O1—H2O1	129.9
C11—N11—Ni1	120.2 (2)	H1O1—O1—H2O1	100.9
C15—N11—Ni1	122.2 (2)		

Symmetry code: (i) −*x*+1, −*y*+1, −*z*+1.

### Diaquabis(pyridine-4-carbonitrile)dithiocyanatonickel(II) Hydrogen-bond geometry (Å, º)

**Table:**

*D*—H···*A*	*D*—H	H···*A*	*D*···*A*	*D*—H···*A*
O1—H1*O*1···S1^ii^	0.84	2.48	3.307 (3)	167
O1—H2*O*1···S1^iii^	0.84	2.44	3.225 (3)	156
C11—H11···N12^iv^	0.95	2.44	3.129 (6)	129

Symmetry codes: (ii) −*x*+1, *y*−1/2, −*z*+3/2; (iii) −*x*+1, −*y*+1, −*z*+2; (iv) −*x*+2, *y*−1/2, −*z*+3/2.

## References

[bb1] Braga, D., Curzi, M., Grepioni, F. & Polito, M. (2005). *Chem. Commun.* pp. 2915–2917. 10.1039/b503404c 15957024

[bb2] Braga, D., Giaffreda, S. L., Grepioni, F., Pettersen, A., Maini, L., Curzi, M. & Polito, M. (2006). *Dalton Trans.* pp. 1249–1263. 10.1039/b516165g 16505902

[bb3] Brandenburg, K. (1999). *DIAMOND*. Crystal Impact GbR, Bonn, Germany.

[bb4] Bruno, I. J., Cole, J. C., Edgington, P. R., Kessler, M., Macrae, C. F., McCabe, P., Pearson, J. & Taylor, R. (2002). *Acta Cryst.* B**58**, 389–397. 10.1107/s0108768102003324 12037360

[bb5] Chen, W., Liu, F. & You, X. (2002). *Bull. Chem. Soc. Jpn* **75**, 1559–1560.

[bb6] Clegg, W. & Harrington, R. W. (2016). CSD Communication, Refcode UBUBOL.

[bb7] Do, J. L. & Friščić, T. (2017). *ACS Cent. Sci.* **3**, 13–19. 10.1021/acscentsci.6b00277 PMC5269651 28149948

[bb8] Ferrando-Soria, J., Vallejo, J., Castellano, M., Martínez-Lillo, J., Pardo, E., Cano, J., Castro, I., Lloret, F., Ruiz-García, R. & Julve, M. (2017). *Coord. Chem. Rev.* **339**, 17–103.

[bb9] Groom, C. R., Bruno, I. J., Lightfoot, M. P. & Ward, S. C. (2016). *Acta Cryst.* B**72**, 171–179. 10.1107/S2052520616003954 PMC4822653 27048719

[bb10] Handy, J. V., Ayala, G. & Pike, R. D. (2017). *Inorg. Chim. Acta* **456**, 64–75.

[bb11] Höller, C. J. & Müller-Buschbaum, K. (2008). *Inorg. Chem.* **47**, 10141–10149. 10.1021/ic800635u 18841934

[bb12] James, S. L., Adams, C. J., Bolm, C., Braga, D., Collier, P., Friščić, T., Grepioni, F., Harris, K. D. M., Hyett, G., Jones, W., Krebs, A., Mack, J., Maini, L., Orpen, A. G., Parkin, I. P., Shearouse, W. C., Steed, J. W. & Waddell, D. (2012). *Chem. Soc. Rev.* **41**, 413–447. 10.1039/c1cs15171a 21892512

[bb13] Jochim, A., Jess, I. & Näther, C. (2017). *Acta Cryst.* E**73**, 463–466. 10.1107/S205698901700322X PMC5382599 28435698

[bb14] Mautner, F. E., Traber, M., Fischer, R. C., Torvisco, A., Reichmann, K., Speed, S., Vicente, R. & Massoud, S. S. (2018). *Polyhedron* **154**, 436–442.

[bb15] Müller-Buschbaum, K. (2005). *Z. Anorg. Allg. Chem.* **631**, 811–828.

[bb16] Näther, C. & Greve, J. (2003). *J. Solid State Chem.* **176**, 259–265.

[bb17] Neumann, T., Ceglarska, M., Germann, L. S., Rams, M., Dinnebier, R. E., Suckert, S., Jess, I. & Näther, C. (2018). *Inorg. Chem.* **57**, 3305–3314. 10.1021/acs.inorgchem.8b00092 29505252

[bb18] Rams, M., Jochim, A., Böhme, M., Lohmiller, T., Ceglarska, M., Rams, M. M., Schnegg, A., Plass, W. & Näther, C. (2020). *Chem. Eur. J.* **26**, 2837–2851. 10.1002/chem.201903924 PMC7078958 31702081

[bb19] Sheldrick, G. M. (2008). *Acta Cryst.* A**64**, 112–122. 10.1107/S0108767307043930 18156677

[bb20] Sheldrick, G. M. (2015*a*). *Acta Cryst.* A**71**, 3–8.

[bb21] Sheldrick, G. M. (2015*b*). *Acta Cryst.* C**71**, 3–8.

[bb22] Stoe (2008). *X-AREA*, *X-RED32* and *X-SHAPE*. Stoe & Cie, Darmstadt, Germany.

[bb23] Stolar, T., Batzdorf, L., Lukin, S., Žilić, D., Motillo, C., Friščić, T., Emmerling, F., Halasz, I. & Užarević, K. (2017). *Inorg. Chem.* **56**, 6599–6608. 10.1021/acs.inorgchem.7b00707 28537382

[bb24] Suckert, S., Rams, M., Böhme, M., Germann, L. S., Dinnebier, R. E., Plass, W., Werner, J. & Näther, C. (2016). *Dalton Trans.* **45**, 18190–18201. 10.1039/c6dt03752f 27796392

[bb25] Wellm, C., Neumann, T., Gallo, G., Dziubyna, A. M., Rams, M., Dinnebier, R. E. & Näther, C. (2020). *Cryst. Growth Des.* **20**, 3374–3385.

[bb26] Werner, J., Rams, M., Tomkowicz, Z. & Näther, C. (2014). *Dalton Trans.* **43**, 17333–17342. 10.1039/c4dt02271h 25318637

[bb27] Westrip, S. P. (2010). *J. Appl. Cryst.* **43**, 920–925.

[bb28] Wöhlert, S., Ruschewitz, U. & Näther, C. (2012). *Cryst. Growth Des.* **12**, 2715–2718.

[bb29] Wöhlert, S., Wriedt, M., Fic, T., Tomkowicz, Z., Haase, W. & Näther, C. (2013). *Inorg. Chem.* **52**, 1061–1068. 10.1021/ic302370n 23276282

[bb30] Wriedt, M. & Näther, C. (2010). *Chem. Commun.* **46**, 4707–4709. 10.1039/c0cc00064g 20583337

[bb31] Yue, G. & Gao, E. Q. (2019). *Coord. Chem. Rev.* **382**, 1–31.

[bb32] Zurawski, A., Rybak, J. C., Meyer, L. V., Matthes, P. R., Stepanenko, V., Dannenbauer, N., Würthner, F. & Müller-Buschbaum, K. (2012). *Dalton Trans.* **41**, 4067–4078. 10.1039/c2dt12047j 22261989

